# Supplementary material for: An Epidemiological Systematic Review with Meta-Analysis on Biomarker Role of Circulating MicroRNAs in Breast Cancer Incidence
Source: Int J Mol Sci. 2023 Feb 15;24(4):3910. doi: 10.3390/ijms24043910 (PMC9967215; doi:10.3390/ijms24043910)
Supplement: Supplementary file 1 [file ijms-24-03910-s001.zip › ijms-2164235-supplementary.pdf]

Supplementary Files

Supplementary Figure S1. Forest plot of included studies assessing the sensitivity and specificity of serum specimen and summary receiver operating characteristic curve (SROC) of MIR34a in breast cancer diagnosis (squares shows sensitivity and specificity, respectively; red diamonds shows pooled effect; error bars represents 95% confidence interval).

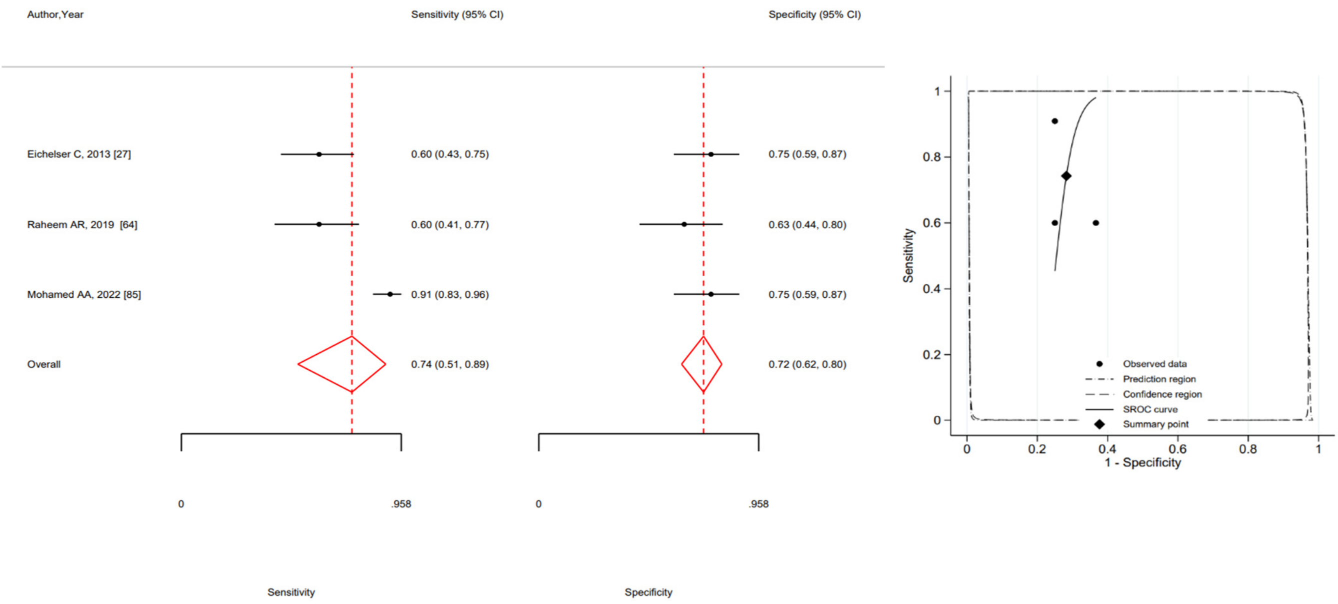

Supplementary Figure S2. Forest plot of included studies assessing the sensitivity and specificity of serum specimen and summary receiver operating characteristic curve (SROC) of MIR195 in breast cancer diagnosis (squares shows sensitivity and specificity, respectively; red diamonds shows pooled effect; error bars represents 95% confidence interval).

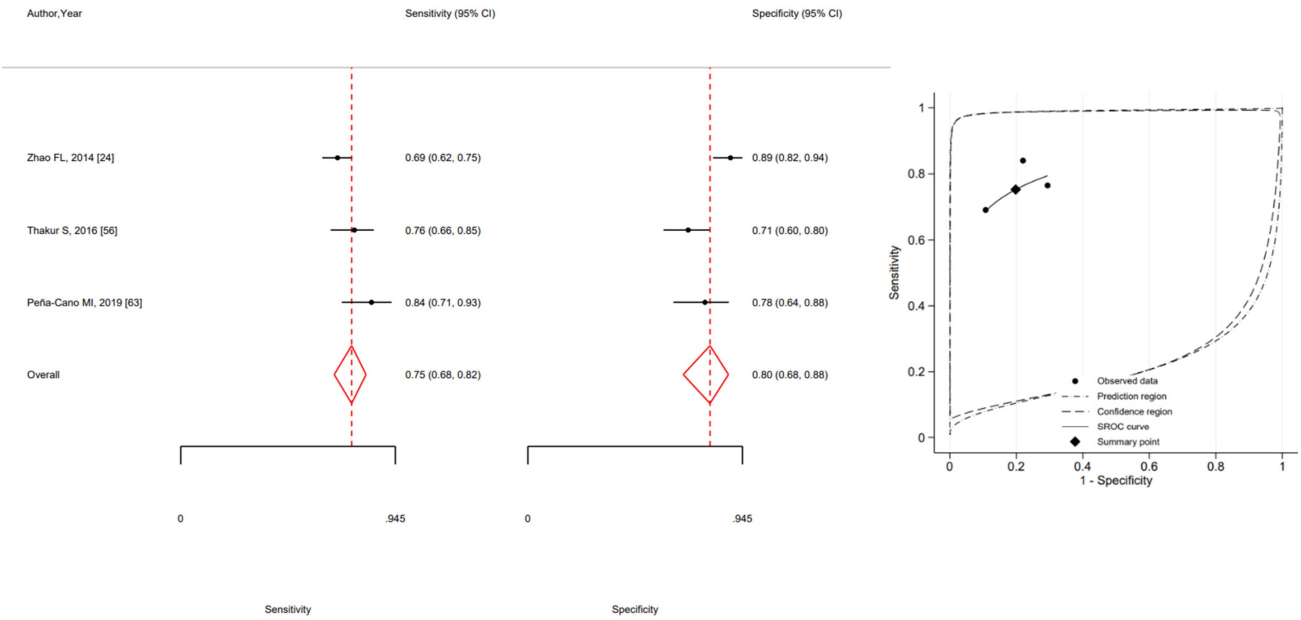

Supplementary Table S1. Clinical characteristics of the breast cancer cases.

**First Author, Year**	**Hystological Subtype**	**Tumor Size (pT)**	**Metastasis**	**Hormonal subtype (positive %)**
Zhu W, 2009 [13]	Ductal:<br>Lobular:<br>Other:	IS<br>T1+T2:<br>>T2:	M0:<br>M1:	Estrogen rec 77%<br>Progesteron rec 69%<br>Her2 rec 8%
Heneghan H, 2010 [14]	Ductal: 71%<br>Lobular:<br>Other:	IS 12%<br>T1+T2: 71%<br>>T2:	M0:<br>M1:	Estrogen rec<br>Progesteron rec<br>Her2 rec
Roth C, 2010 [15]	Ductal:<br>Lobular:<br>Other:	IS 12%<br>T1+T2: 71%<br>>T2:	M0: 100%	Estrogen rec<br>Progesteron rec<br>Her2 rec
Wang F, 2010 [16]	Ductal: 85%<br>Lobular:<br>Other:	IS<br>T1+T2: 48%<br>>T2:	M0:<br>M1:	Estrogen rec 59%<br>Progesteron rec 56%<br>Her2 rec
Asaga S, 2011 [17]	Ductal:<br>Lobular:<br>Other:	IS<br>T1+T2:<br>>T2:	M0:<br>M1: 21%	Estrogen rec<br>Progesteron rec<br>Her2 rec
Guo LJ, 2012 [18]	Ductal:<br>Lobular:<br>Other:	IS<br>T1+T2:<br>>T2:	M0:<br>M1:	Estrogen rec 68%<br>Progesteron rec 50%<br>Her2 rec
Schrauder MG, 2012 [19]	Ductal:<br>Lobular:<br>Other:	IS<br>T1+T2: 24%<br>>T2:	M0:<br>M1:	Estrogen rec 87%<br>Progesteron rec 67%<br>Her2 rec 4%
Schwarzenbach H, 2012 [20]	Ductal: 63%<br>Lobular: 21%<br>Other: 16%	IS<br>T1: 44%<br>≥T2: 56%	M0:<br>M1:	Estrogen rec 80%<br>Progesteron rec 66%<br>Her2 rec
Sun Y, 2012 [21]	Ductal:<br>Lobular:<br>Other:	IS<br>T1+T2: 52%<br>>T2: 48%	M0:<br>M1:	Estrogen rec 67%<br>Progesteron rec 65%<br>Her2 rec 21%
van Schooneveld E, 2012 [22]	Ductal:<br>Lobular:	IS<br>T1+T2: 70%	M0:<br>M1: 95%	Estrogen rec 69%<br>Progesteron rec 52%

	Other:	>T2: 30%		Her2 rec 35%
Wu Q, 2012 [23]	Ductal:<br>Lobular:<br>Other:	IS<br>T1+T2:<br>>T2:	M0:<br>M1:	Estrogen rec<br>Progesteron rec<br>Her2 rec
Zhao FL, 2012 [24]	Ductal:<br>Lobular:<br>Other:	IS<br>T1+T2:<br>>T2:	M0:<br>M1:	Estrogen rec<br>Progesteron rec<br>Her2 rec
Chan M, 2013 [25]	Ductal:<br>Lobular:<br>Other:	IS: 2%<br>T1+T2: 92%<br>>T2: 6%	M0: 92%<br>M1: 8%	Estrogen rec<br>Progesteron rec<br>Her2 rec
Cuk K, 2013 [26]	Ductal: 78%<br>Lobular: 9%<br>Other: 13%	IS: 3%<br>T1: 47%<br>≥T2: 50%	M0:<br>M1:	Estrogen rec<br>Progesteron rec<br>Her2 rec
Eichelser C, 2013 [27]	Ductal: 75%<br>Lobular:15%<br>Other:	IS<br>T1: 58%<br>≥T2: 42%	M0: 100%<br>M1:	Estrogen rec<br>Progesteron rec<br>Triple negative: 33%
Godfrey AC, 2013 [28]	Ductal:<br>Lobular:<br>Other:	IS<br>T1+T2:<br>>T2:	M0:<br>M1:	Estrogen rec<br>Progesteron rec<br>Her2 rec
Kumar S, 2013 [29]	Ductal:<br>Lobular:<br>Other:	IS<br>T1+T2:<br>>T2:	M0:<br>M1:	Estrogen rec<br>Progesteron rec<br>Her2 rec
Ng EKO, 2013 [30]	Ductal:<br>Lobular:<br>Other:	IS : 16%<br>T1+T2: 62%<br>>T2: 22%	M0:<br>M1:	Estrogen rec<br>Progesteron rec<br>Her2 rec
Si H, 2013 [31]	Ductal:<br>Lobular:<br>Other:	IS<br>T1+T2:<br>>T2:	M0:<br>M1:	Estrogen rec<br>Progesteron rec<br>Her2 rec
Wang PY, 2013 [32]	Ductal:<br>Lobular:<br>Other:	IS<br>T1+T2:<br>>T2:	M0:<br>M1:	Estrogen rec<br>Progesteron rec<br>Her2 rec
Zeng RC, 2013 [33]	Ductal:<br>Lobular:	IS : 6%<br>T1+T2: 87%	M0:<br>M1:	Estrogen rec: 77%<br>Progesteron rec: 87%

	Other:	>T2: 17%		Her2 rec 17%
Eichelser C, 2014 [34]	Ductal: 65%<br>Lobular: 19%<br>Other: 16%	IS<br>T1+T2:<br>>T2:	M0:<br>M1:	Estrogen rec:<br>Progesteron rec:<br>Her2 rec:
Hamdi K, 2014 [35]	Ductal:<br>Lobular:<br>Other:	IS<br><T4:<br>T4: 100%	M0:<br>M1:	Estrogen rec: 15%<br>Progesteron rec: 20%<br>Her2 rec: 65%
Joosse SA, 2014 [36]	Ductal: 63%<br>Lobular:21%<br>Other: 16%	IS<br>T1+T2: 44%<br>>T2: 56%	M0:<br>M1:	Estrogen rec 80%<br>Progesteron rec 64%<br>Her2 rec
Kodahl AR, 2014 [37]	Ductal:<br>Lobular:<br>Other:	IS<br>T1+T2: 100%<br>>T2:	M0: 100%<br>M1:	Estrogen rec 10%<br>Progesteron rec 70%<br>Her2 rec
Mar-Aguilar F, 2014 [38]	Ductal:<br>Lobular:<br>Other:	IS<br>T1+T2:<br>>T2:	M0:<br>M1:	Estrogen rec:<br>Progesteron rec:<br>Her2 rec:
McDermott AM, 2014 [39]	Ductal: 97%<br>Lobular:3%<br>Other:	IS<br>T1+T2:<br>>T2:	M0:<br>M1:	Estrogen rec 100%<br>Progesteron rec 100%<br>Her2 rec 0%
Shen J, 2014 [40]	Ductal:<br>Lobular:<br>Other:	IS<br>T1+T2:<br>>T2:	M0:<br>M1:	Estrogen rec<br>Progesteron rec<br>Her2 rec
Sochor M, 2014 [41]	Ductal:<br>Lobular:<br>Other:	IS<br>T1+T2:<br>>T2:	M0:<br>M1:	Estrogen rec<br>Progesteron rec<br>Her2 rec
Zearo S, 2014 [42]	Ductal: 91%<br>Lobular:9%<br>Other:	IS<br>T1+T2:<br>>T2:	M0:<br>M1:	Estrogen rec<br>Progesteron rec<br>Her2 rec
Zhao FL, 2014 [43]	Ductal: 91%<br>Lobular:3%<br>Other:	IS: 28%<br>T1+T2: 67%<br>>T2: %	M0:<br>M1:	Estrogen rec 66%<br>Progesteron rec 58%<br>Her2 rec
Antolin S, 2015 [44]	Ductal:<br>Lobular:	IS<br>T1+T2:	M0:<br>M1:	Estrogen rec<br>Progesteron rec

	Other:	>T2:		Triple neg: 10%
Li XX, 2015 [45]	Ductal:<br>Lobular:<br>Other:	IS<br>T1+T2:<br>>T2:	M0:<br>M1:	Estrogen rec<br>Progesteron rec<br>Her2 rec
Mangolini A (A), 2015 [46]	Ductal: 75%<br>Lobular:14%<br>Other:	IS<br>T1+T2:<br>>T2:	M0: 100%<br>M1:	Estrogen rec 66%<br>Progesteron rec 58%<br>Her2 rec
Mangolini A (B), 2015 [46]	Ductal: 81%<br>Lobular: 7%<br>Other:	IS:<br>T1+T2: 93%<br>>T2: 7%	M0: 97%<br>M1: 3%	Estrogen rec 69%<br>Progesteron rec 59%<br>Her2 rec
Matamala N, 2015 [47]	Ductal:<br>Lobular:<br>Other:	IS<br>T1+T2:<br>>T2:	M0:<br>M1:	Estrogen rec<br>Progesteron rec<br>Her2 rec
Shaker O, 2015 [48]	Ductal: 85%<br>Lobular:5%<br>Other: 10%	IS<br>T2: 74%<br>T3: 26%	M0: 80%<br>M1: 20%	Estrogen rec:<br>Progesteron rec:<br>Her2 rec:
Zhang L, 2015 [49]	Ductal:<br>Lobular:<br>Other:	IS<br>T1+T2:<br>>T2:	M0:<br>M1:	Estrogen rec 41%<br>Progesteron rec 30%<br>Her2 rec 25%
Frères P, 2016 [50]	Ductal:<br>Lobular:<br>Other:	IS<br>T1: 42%<br>≥T2: 58%	M0: 100%<br>M1:	Estrogen rec 79%<br>Progesteron rec 73%<br>Her2 rec 20%
Fu L, 2016 [51]	Ductal:<br>Lobular:<br>Other:	IS<br>T1+T2:<br>>T2:	M0:<br>M1: +T2	Estrogen rec: 54%<br>Progesteron rec: 46%<br>Her2 rec: 56%
Hamam R, 2016 [52]	Ductal:<br>Lobular:<br>Other:	IS<br>T1+T2:<br>>T2:	M0:<br>M1:	Estrogen rec<br>Tripl neg: 15%<br>Her2 rec 37%
Hannafon BN, 2016 [53]	Ductal:<br>Lobular:<br>Other:	IS<br>T1+T2:<br>>T2:	M0:<br>M1:	Estrogen rec 81%<br>Progesteron rec 75%<br>Her2 rec 19%
Motawi TM, 2016 [54]	Ductal: 92%<br>Lobular: 8%	IS<br>T2: 70%	M0:<br>M1:	Estrogen rec 17%<br>Progesteron rec

	Other:	T3: 15%		Her2 rec
Shimomura A, 2016 [55]	Ductal:<br>Lobular:<br>Other:	IS<br>T1+T2:<br>>T2:	M0:<br>M1:	Estrogen rec<br>Progesteron rec<br>Her2 rec
Thakur S, 2016 [56]	Ductal:<br>Lobular:<br>Other:	IS<br>T1+T2:<br>>T2:	M0:<br>M1:	Estrogen rec<br>Progesteron rec<br>Tripl neg: 27%
Gao S, 2017 [57]	Ductal: 77%<br>Lobular:12%<br>Other: 11%	IS<br>T1+T2: 56%<br>>T2: 24%	M0: 67%<br>M1: 33%	Estrogen rec<br>Progesteron rec<br>Her2 rec
Zhang K, 2017 [58]	Ductal:<br>Lobular:<br>Other:	IS 6%<br>T1+T2: 88%<br>>T2: 6%	M0:<br>M1:	Estrogen rec 87%<br>Progesteron rec 73%<br>Her2 rec 13%
Heydari N, 2018 [59]	Ductal: 80%<br>Lobular:20%<br>Other:	IS: 3%<br>T1+T2: 85%<br>>T2: 12%	M0:<br>M1:	Estrogen rec 87%<br>Progesteron rec 80%<br>Her2 rec 30%
Zaleski M, 2018 [60]	Ductal:<br>Lobular:<br>Other:	IS: 4%<br>T1+T2: 74%<br>>T2: 21%	M0: 89%<br>M1: 11%	Estrogen rec<br>Progesteron rec<br>Her2 rec
Kaharam M, 2019 [61]	Ductal: 100%<br>Lobular:<br>Other:	IS<br>T1+T2:<br>>T2:	M0: 100%<br>M1:	Estrogen rec 100%<br>Progesteron rec 100%<br>Her2 rec
McAnena P, 2019 [62]	Ductal:<br>Lobular:<br>Other:	IS<br>T1+T2:<br>>T2:	M0:<br>M1:	Estrogen rec:<br>Progesteron rec:<br>Her2 rec:
Peña-Cano MI, 2019 [63]	Ductal:<br>Lobular:<br>Other:	IS<br>T1+T2:<br>>T2:	M0:<br>M1:	Estrogen rec<br>Progesteron rec<br>Her2 rec
Raheem AR, 2019 [64]	Ductal: 63%<br>Lobular: 7%<br>Other: 30%	IS.<br>T1: 37%<br>≥T2: 63%	M0:<br>M1:	Estrogen rec 47%<br>Progesteron rec 47%<br>Her2 rec 20%
Soleimanpour E, 2019 [65]	Ductal:<br>Lobular:	IS. 30%<br>T1: 47%	M0:<br>M1:	Estrogen rec:<br>Progesteron rec:

	Other:	≥T2: 50%		Her2 rec:
Anwar SL, 2020 [66]	Ductal:<br>Lobular:<br>Other:	IS<br>T1+T2:<br>>T2:	M0:<br>M1:	Estrogen rec 55%<br>Progesteron rec 55%<br>Her2 rec 19%
Arabkari V, 2020 [67]	Ductal:<br>Lobular:<br>Other:	IS<br>T1+T2:<br>>T2:	M0:<br>M1:	Estrogen rec 100%<br>Progesteron rec 100%<br>Her2 rec 0%
Ashirbekov Y, 2020 [68]	Ductal:<br>Lobular:<br>Other:	IS<br>T1+T2: 91%<br>>T2: 9%	M0:<br>M1: 3%	Estrogen rec 77%<br>Progesteron rec 71%<br>Her2 rec 20%
Guo H, 2020 [69]	Ductal:<br>Lobular:<br>Other:	IS<br>T1: 71%<br>≥T2: 23%	M0:<br>M1:	Estrogen rec 67%<br>Progesteron rec 46%<br>Her2 rec 20%
Holubekova V, 2020 [70]	Ductal:<br>Lobular:<br>Other:	IS: 7%<br>T1+T2: 66%<br>>T2: 27%	M0:<br>M1:	Estrogen rec 87%<br>Progesteron rec 78%<br>Her2 rec 18%
Hosseini Mojahed FH, 2020 [71]	Ductal:<br>Lobular:<br>Other:	IS<br>T1: 30%<br>≥T2: 69%	M0:<br>M1:	Estrogen rec 67%<br>Progesteron rec 53%<br>Her2 rec 25%
Ibrahim AM, 2020 [72]	Ductal:<br>Lobular:<br>Other:	IS<br>T1+T2:<br>>T2:	M0:<br>M1:	Estrogen rec<br>Progesteron rec<br>Her2 rec
Jang JY, 2020 [73]	Ductal:<br>Lobular:<br>Other:	IS<br>T1+T2:<br>>T2:	M0:<br>M1:	Estrogen rec<br>Progesteron rec<br>Her2 rec
Kim J, 2020 [74]	Ductal:<br>Lobular:<br>Other:	IS<br>T1+T2:<br>>T2:	M0:<br>M1:	Estrogen rec<br>Progesteron rec<br>Her2 rec
Pastor-Navarro B, 2020 [75]	Ductal:<br>Lobular:<br>Other:	IS<br>T1+T2:<br>>T2:	M0:<br>M1:	Estrogen rec<br>Progesteron rec<br>Her2 rec
Bakr NM, 2021 [76]	Ductal:<br>Lobular:	IS: 45%<br>T1+T2:	M0:<br>M1:	Estrogen rec: 66%<br>Progesteron rec: 69%

	Other:	>T2:		Her2 rec: 63%
Diansyah MN, 2021 [77]	Ductal:<br>Lobular:<br>Other:	IS<br>T1+T2: 100%<br>>T2:	M0:<br>M1:	Estrogen rec<br>Progesteron rec<br>Her2 rec
Itani MM, 2021 [78]	Ductal:<br>Lobular:<br>Other:	IS<br>T1+T2: 66%<br>>T2: 34%	M0: 100%<br>M1:	Estrogen rec<br>Progesteron rec<br>Her2 rec
Mohmmed EA, 2021 [79]	Ductal: 80%<br>Lobular:10%<br>Other:	IS<br>T1: 16%<br>≥T2: 84%	M0:<br>M1:	Estrogen rec: 40%<br>Progesteron rec: 34%<br>Her2 rec: 26%
Nashtahosseini Z, 2021 [80]	Ductal: 54%<br>Lobular:19%<br>Other:	IS: 5%<br>T1: 9%<br>T2: 91%	M0:<br>M1:	Estrogen rec<br>Progesteron rec<br>Her2 rec
Zhang K, 2021 [81]	Ductal:<br>Lobular:<br>Other:	IS<br>T1+T2:<br>>T2:	M0:<br>M1:	Estrogen rec<br>Progesteron rec<br>Her2 rec
Zhao T, 2021 [82]	Ductal:<br>Lobular:<br>Other:	IS<br>T1+T2:<br>>T2:	M0:<br>M1:	Estrogen rec 41%<br>Progesteron rec 53%<br>Her2 rec 58%
Li X, 2022 [83]	Ductal: 82%<br>Lobular:4%<br>Other:	IS<br>T1+T2:<br>>T2:	M0:<br>M1:	Estrogen rec 61%<br>Progesteron rec 57%<br>Her2 rec 71%
Liu H, 2022 [84]	Ductal:<br>Lobular:<br>Other:	IS<br>T1: 21%<br>≥T2: 79%	M0:<br>M1:	Estrogen rec 68%<br>Progesteron rec 58%<br>Her2 rec 64%
Mohamed AA, 2022 [85]	Ductal:<br>Lobular:<br>Other:	IS<br>T1: 25%<br>≥T2: 75%	M0:91%<br>M1:9%	Estrogen rec 48%<br>Progesteron rec 50%<br>Her2 rec 27%
Zavesky L, 2022 [86]	Ductal:<br>Lobular:<br>Other:	IS<br>T1+T2:<br>>T2:	M0:<br>M1:	Estrogen rec 100%<br>Progesteron rec 100%<br>Her2 rec 0%
Zou R, 2022 [87]	Ductal:<br>Lobular:	IS<br>T1+T2:	M0:<br>M1:	Estrogen rec<br>Progesteron rec

	Other:	>T2:		Her2 rec

Supplementary Table S2. Evaluation of the quality of the studies (QUADAS2).

	Risk of bias			
	**Patient selection**	**Index test**	**Reference standard**	**Flow and timing**
Zhu W, 2009	High	High	High	High
Heneghan H, 2010	Low	High	Low	Low
Roth C, 2010	High	High	High	Low
Wang F, 2010	High	High	Low	Low
Asaga S, 2011	High	Low	High	High
Guo LJ, 2012	High	Low	Low	Low
Schrauder MG, 2012	Low	High	Low	Low
Schwarzenbach H, 2012	High	High	High	Low
Sun Y, 2012	High	Low	Low	Low
van Schooneveld E, 2012	High	High	High	Low
Wu Q, 2012	High	Low	Low	Low
Zhao FL, 2012	High	High	Low	High
Chan M, 2013	High	High	High	High
Cuk K, 2013	High	High	High	Low
Eichelser C, 2013	High	High	Low	High
Godfrey AC, 2013	Low	High	Low	Low
Kumar S, 2013	High	High	High	High
Ng EKO, 2013	High	High	High	Low
Si H, 2013	High	High	High	High
Wang PY, 2013	High	High	Low	Low
Zeng RC, 2013	High	Low	Low	Low
Eichelser C, 2014	High	High	Low	Low
Hamdi K, 2014	High	High	High	High
Joosse SA, 2014	High	High	High	Low
Kodahl AR, 2014	Low	High	Low	Low
Mar-Aguilar F, 2014	High	Low	High	High
McDermott AM, 2014	Low	High	Low	Low
Shen J, 2014	High	High	Low	Low
Sochor M, 2014	High	High	Low	Low
Zearo S, 2014	High	High	High	High
Zhao FL, 2014	Low	Low	Low	Low
Antolin S, 2015	Low	High	Low	Low
Li XX, 2015	High	High	Low	Low
Mangolini A (A), 2015	High	High	High	High
Mangolini A (B), 2015	High	High	High	High
Matamala N, 2015	High	High	High	High
Shaker O, 2015	High	Low	Low	High
Zhang L, 2015	High	High	High	High
Frères P, 2016	High	High	High	Low
Fu L, 2016	High	Low	Low	High
Hamam R, 2016	High	High	High	High

Hannafon BN, 2016	High	High	High	High
Motawi TM, 2016	High	Low	Unclear	Low
Shimomura A, 2016	High	High	Unclear	Low
Thakur S, 2016	High	High	Low	Low
Gao S, 2017	High	High	High	High
Zhang K, 2017	High	Low	Low	High
Heydari N, 2018	High	Low	High	High
Zaleski M, 2018	High	High	High	Low
Kaharam M, 2019	Low	High	Unclear	Low
McAnena P, 2019	Low	High	Low	Low
Peña-Cano MI, 2019	High	Low	Low	High
Raheem AR, 2019	High	Low	High	Low
Soleimanpour E, 2019	High	High	Low	High
Anwar SL, 2020	High	High	High	High
Arabkari V, 2020	High	High	High	High
Ashirbekov Y, 2020	High	High	High	High
Guo H, 2020	High	High	High	Low
Holubekova V, 2020	High	High	High	High
Hosseini Mojahed FH, 2020	High	Low	Low	High
Ibrahim AM, 2020	High	Low	Low	Low
Jang JY, 2020	High	High	High	Low
Kim J, 2020	High	Low	Low	High
Pastor-Navarro B, 2020	High	High	Low	Low
Bakr NM, 2021	High	Low	High	High
Diansyah MN, 2021	Low	Low	High	High
Itani MM, 2021	Unclear	Low	High	High
Mohmmed EA, 2021	Low	High	High	Low
Nashtahosseini Z, 2021	Low	Low	Low	Low
Zhang K, 2021	Low	Low	Low	Low
Zhao T, 2021	High	High	High	High
Li X, 2022	Low	High	Low	Low
Liu H, 2022	Low	Low	Low	Low
Mohamed AA, 2022	High	Low	High	Low
Zavesky L, 2022	High	High	Low	Low
Zou R, 2022	Unclear	High	High	High
	Patient selection	Index test	Reference standard	Flow and timing
Low	15	24	35	42
High	59	52	38	34
Unclear	2	0	3	0

APPLICABILITY ASSESSMENT			
	Patient selection	Index test	Reference standard
Zhu W, 2009	Low	Low	High
Heneghan H, 2010	Low	Low	Low
Roth C, 2010	Low	Low	High
Wang F, 2010	Low	Low	Low
Asaga S, 2011	Low	Low	High
Guo LJ, 2012	Low	Low	Low
Schrauder MG, 2012	Low	Low	Low
Schwarzenbach H, 2012	High	Low	Low
Sun Y, 2012	Low	Low	Low
van Schooneveld E, 2012	High	Low	High
Wu Q, 2012	Low	Low	Low
Zhao FL, 2012	Low	Low	Low
Chan M, 2013	Low	Low	High
Cuk K, 2013	Low	Low	High
Eichelser C, 2013	High	Low	Low
Godfrey AC, 2013	Low	Low	Low
Kumar S, 2013	Low	Low	High
Ng EKO, 2013	Low	Low	High
Si H, 2013	Low	Low	High
Wang PY, 2013	Low	Low	High
Zeng RC, 2013	Low	Low	Low
Eichelser C, 2014	High	Low	Low
Hamdi K, 2014	High	Low	High
Joosse SA, 2014	Low	Low	High
Kodahl AR, 2014	Low	Low	Low
Mar-Aguilar F, 2014	Low	Low	High
McDermott AM, 2014	High	Low	Low
Shen J, 2014	Low	Low	Low
Sochor M, 2014	Low	Low	Low
Zearo S, 2014	Low	Low	High
Zhao FL, 2014	Low	Low	Low
Antolin S, 2015	Low	Low	Low
Li XX, 2015	Low	Low	Low
Mangolini A (A), 2015	Low	Low	High
Mangolini A (B), 2015	Low	Low	High
Matamala N, 2015	Low	Low	High
Shaker O, 2015	Low	Low	High
Zhang L, 2015	Low	Low	High
Frères P, 2016	Low	Low	High
Fu L, 2016	Low	Low	Low
Hamam R, 2016	Low	Low	High
Hannafon BN, 2016	Low	Low	High
Motawi TM, 2016	Low	Low	Unclear
Shimomura A, 2016	Low	Low	Unclear
Thakur S, 2016	Low	Low	Low

Gao S, 2017	Low	Low	High
Zhang K, 2017	Low	Low	Low
Heydari N, 2018	Low	Low	High
Zaleski M, 2018	Low	Low	High
Kaharam M, 2019	High	Low	Low
McAnena P, 2019	High	Low	Low
Peña-Cano MI, 2019	High	Low	Low
Raheem AR, 2019	Low	Low	High
Soleimanpour E, 2019	Low	Low	Low
Anwar SL, 2020	Low	Low	High
Arabkari V, 2020	High	Low	High
Ashirbekov Y, 2020	Low	Low	High
Guo H, 2020	Low	Low	High
Holubekova V, 2020	Low	Low	High
Hosseini Mojahed FH, 2020	Low	Low	Low
Ibrahim AM, 2020	High	Low	Low
Jang JY, 2020	Low	Low	High
Kim J, 2020	Low	Low	High
Pastor-Navarro B, 2020	Low	Low	High
Bakr NM, 2021	High	Low	High
Diansyah MN, 2021	Low	Low	Low
Itani MM, 2021	High	Low	High
Mohmmed EA, 2021	Low	Low	Low
Nashtahosseini Z, 2021	Low	Low	Low
Zhang K, 2021	Low	Low	Low
Zhao T, 2021	Low	Low	High
Li X, 2022	Low	Low	Low
Liu H, 2022	Unclear	Low	Low
Mohamed AA, 2022	Low	Low	Low
Zavesky L, 2022	High	Low	High
Zou R, 2022	Unclear	Low	High
Low	60	76	35
High	14	0	39
Unclear	2	0	2
